# Prospective Analysis of ^18^F-FDG PET/CT Predictive Value in Patients with Low Rectal Cancer Treated with Neoadjuvant Chemoradiotherapy and Conservative Surgery

**DOI:** 10.1155/2014/952843

**Published:** 2014-05-04

**Authors:** Artor Niccoli-Asabella, Corinna Altini, Raffaele De Luca, Margherita Fanelli, Domenico Rubini, Cosimo Caliandro, Severino Montemurro, Giuseppe Rubini

**Affiliations:** ^1^Nuclear Medicine Unit, DIM, University of Bari “Aldo Moro”, Piazza G. Cesare 11, 70124 Bari, Italy; ^2^Department of Surgical Oncology, Istituto Tumori “G. Paolo II”, Viale Orazio Flacco 65, 70124 Bari, Italy

## Abstract

This study prospectively assessed ^18^F-FDG PET/CT in predicting the response of locally advanced low rectal cancer (LRC) to neoadjuvant chemoradiation (nCRT). *Methods*. 56 patients treated with chemoradiation underwent two ^18^F-FDG PET/CT scans (baseline and 5-6 weeks post-nCRT). ^18^F-FDG uptake (SUVmax and SUVmean) and differences between baseline (SUV1) and post-nCRT (SUV2) scans (ΔSUV and RI%) were evaluated. Results were related to the Mandard's TRG and (y)pTNM. *Results*. ^18^F-FDG PET/CT sensitivity, specificity, accuracy, PPV and NPV resulted in 88.6%, 66.7%, 83.92%, 90.7%, and 61.5%. SUV2 resulted in better than SUV1 to predict nCRT response by TRG, with no significant statistical difference between the SUVmax2 and SUVmean2 AUC (0.737 versus 0.736; *P* = 0.928). The same applies to the (y)pTNM (0.798 versus 0.782; *P* = 0.192). In relation to the TRG, RI values had a higher AUC than ΔSUV, with no significant difference between RImax and RImean (0.672 versus 0.695; *P* = 0.292). The same applied to the (y)pTNM (0.742 versus 0.741; *P* = 0.940). In both cases ΔSUV does not appear to be a good predictive tool. Logistic regression confirmed the better predictive role of SUVmax2 for the (y)pTNM (odds ratio = 1.58) and SUVmean2 for the TRG (odds ratio = 1.87). *Conclusions*. ^18^F-FDG PET/CT can evaluate response to nCRT in LRC, even if more studies are required to define the most significant parameter for predicting pathologic tumor changes.

## 1. Introduction

Low rectal cancer (LRC) can benefit from neoadjuvant chemoradiotherapy (nCRT) treatments for downstaging purposes [1, 2]. nCRT regimens in patients with locally advanced LRC are intended to control pelvic disease and to improve the chance of sphincter preservation at subsequent surgery, thereby improving overall survival [3–5]. In addition to downstaging the tumor, nCRT produces a complete pathologic response and improves survival in selected patients [6, 7]. Therefore, it is essential to accurately identify responders and nonresponders following nCRT for patients with LRC. Surgery is the fundamental curative approach for LRC [8].

Therefore, in the light of the good prognosis in patients with a complete pathologic response, new more conservative treatment strategies are being developed to avoid rectal resection. This provides many advantages, with a consequent reduction in morbidity and mortality as well as the preservation of the sphincter apparatus [8].

The conventional imaging modalities, including endorectal ultrasound (ERUS), computed tomography (CT), and magnetic resonance (MRI), which have been confirmed as indispensable tests for staging these patients, are unable to differentiate postradiation fibrotic changes from the residual tumor or predict the pathologic response [8–10].

When applied to assess tumor response to nCRT, purely morphological imaging methods can yield equivocal results, overestimating the local tumor extension [11]. On the other hand, the persistence of a gross mass due to fibrosis and edema following irradiation may lead to underestimation of the efficacy of treatment.

The role of 18-fluorine-labeled-2-deoxy-2-fluoro-D-glucose positron-emission-tomography/computed tomography (^18^F-FDG PET/CT) for the prediction of tumor response to different types of therapy is still under debate [12, 13]. More recently, the metabolic information provided by ^18^F-FDG PET/CT has been reported to be capable of more reliably predicting the response to nCRT than the tumor morphology [9, 10, 14, 15].

The issue of how to accurately assess changes in the ^18^F-FDG metabolism during therapy is still under debate, being closely related to different analysis methods. In fact, at the current state of the art, it is not a standard method for LRC.

Functional imaging with ^18^F-FDG has proven to be capable of reliably predicting treatment response. The degree of ^18^F-FDG uptake reductions after neoadjuvant treatment as compared to the baseline value in the pretreatment stage has been proposed as an index for the early prediction of regression in tumors treated with nCRT [8].

The primary endpoint of the present study was to evaluate the role of ^18^F-FDG PET/CT procedures in predicting nCRT response in patients with LRC. The secondary endpoint was to identify standardized ^18^F-FDG PET/CT parameters that are capable of differentiating responders from nonresponders.

We hypothesized that ^18^F-FDG PET/CT can predict the nCRT response and possibly a complete pathologic response. It may be a significant variable that can be applied in patients whose therapeutic approach could be modified to consist of more conservative or less invasive therapy.

## 2. Methods

### 2.1. Patients

We analyzed 56 patients (18 females and 38 males) with a mean age of 62.25 years (range: 35–86 years) and an initial diagnosis of LRC, located less than 8 cm from the anal verge.

All patients underwent conventional diagnostic/staging procedures for characterizing the rectal lesion (location and size, distance from the sphincter apparatus, circumferential resection margin, relationship with neighboring organs, infiltration of the mesorectum, and the existence of adenopathies) with the usual techniques of rectal examination, ERUS, pelvic CT or MR, and colon/rectosigmoidoscopy.

All patients had a biopsy-proven rectal adenocarcinoma. The location of the tumor was defined as the distance between the lower edge of the tumor and the anal verge, and this was measured by a digital examination and a rigid proctoscopy. Tumor characteristics at the moment of initial staging are reported in Table 1.

The following exclusion criteria were applied: pregnancy, age younger than 18 years, previous rectal treatment (chemotherapy, radiotherapy, or surgery), presence of distant metastases at the time of diagnosis, neoadjuvant therapy contraindications due to comorbidity, and/or the presence of another synchronic tumor. Written informed consent was obtained from all patients before enrolment in the study.

The usual techniques of rectal examination (ERUS, pelvic CT or MR, and colon/rectosigmoidoscopy) were repeated at the end of nCRT.

### 2.2. Neoadjuvant Treatments

Chemotherapy, consisting of 5-fluorouracil (435 mg/m^2^/d) and leucovorin (20 mg/m^2^/d) for 32–34 days, was intravenously administered. The whole pelvic field received 25 fractions of 180cGy/d over 5 weeks, for a total of 5040 cGy, using a 4-field box technique. Neoadjuvant chemotherapy was started concurrently on the first day of radiotherapy.

### 2.3. Surgery

All patients were scheduled to undergo surgery 8 weeks after completion of the nCRT. All patients were operated by the same surgical team and received mechanical bowel preparation. In all the operations total mesorectum excision was performed according to Heald's technique.

### 2.4. ^18^F-FDG PET/CT

The first whole-body ^18^F-FDG PET/CT was performed 1 week before beginning the nCRT (baseline scan), to rule out metastatic disease and provide confirmation of the primary tumor. The second ^18^F-FDG PET/CT was scheduled after 5-6 weeks from nCRT completion (post-nCRT scan) in order to avoid potential false-negative results related to chemotherapy or false-positive results related to radiotherapy.

Images were acquired with a combined modality PET/CT Discovery LSA (GE Healthcare, Waukesha, Wisconsin, USA) that integrates a PET (Advance NxI) with 16-slice CT scanner (Light Speed Plus). Prior to administration of ^18^F-FDG, all patients fasted for at least 8 h and had a capillary blood glucose of <160 mg/mL and, to avoid artifacts caused by muscles, they were instructed not to do any physical activity before the examination. The image acquisition was obtained 50 min after the intravenous injection of 4.6 MBq/kg of ^18^F-FDG.

Patients were hydrated by drinking 500 mL of water and urinated. No muscle relaxant drugs were administered. The scan was carried out from the external acoustic meatus to the root of the thigh with patients lying on their back with hands above their head. The CT acquisition parameters were 340 mA (auto), 120 kV, slice thickness 3.75 mm, tube rotation time 0.8 ms, and collimation field of view (FOV) 50 cm. The CT images were reconstructed with a filtered backprojection. The CT data were used for attenuation correction of PET scanning, which was performed immediately after the acquisition of CT images. The CT scans were obtained without administration of contrast medium. The PET acquisition was obtained in caudal-cranial direction; PET was reconstructed with a matrix of 128 × 128, ordered subset expectation maximum iterative reconstruction algorithm (two iterations, 28 subsets), 8 mm Gaussian filter, and 50 cm field of view.

### 2.5. Image Analysis

Two nuclear medicine physicians with 8 years of experience blindly and independently analyzed data at a dedicated XelerisWorkstation (GE Healthcare, Waukesha, Wisconsin, USA).

Regions of interest (ROIs) were drawn on the area of abnormal ^18^F-FDG uptake corresponding to the tumor in the baseline scan and then carefully placed in the identical position and at the same size on the post-nCRT scan, with the aid of the anatomical landmarks provided by CT and fusion PET/CT images, to calculate standardized uptake values (SUV).

SUVmax and SUVmean were calculated using the maximum and mean activity values within each ROI on the transaxial slices with the highest radioactivity concentration, normalized to the injected dose and patient's body weight.

The SUVs values on the baseline scan (SUV1) and the post-nCRT scan (SUV2) to assess tumor response to therapy were employed as follows:

- by calculating the absolute SUV1**−**SUV2 difference (**Δ**SUV),
- by calculating a response index (RI), as RI = [(SUV1**−**SUV2)/SUV1] × 100.

**Δ**SUV was calculated both for SUVmax and SUVmean (**Δ**SUVmax and** Δ**SUVmean) as well as RI (RImax and RImean).

### 2.6. Response Evaluation-Histopathology

All resection specimens were examined by 2 experienced gastrointestinal pathologists. The assessment of the tumor response to nCRT was performed according to Mandard's tumor regression grade (TRG score) [16] and also performed by the evaluation of the (y)pTNM categories according to the International Union against Cancer (UICC, 7th edition, 2010). According to the TRG the patients were divided into two groups: responders (TRG I and II) and nonresponders (TRG III to V), while according to the T parameter of (y)pTNM patients were divided into complete responders (T0) and partial/nonresponders (T1–3).

### 2.7. Statistical Analysis

Sensitivity, specificity, accuracy, positive predictive value (PPV), and negative predictive value (NPV) of post-nCRT ^18^F-FDG PET/CT were evaluated.

The neoadjuvant response was analyzed by evaluating the result of the post-nCRT ^18^F-FDG PET/CT scan (SUVmax2, SUVmean2,** Δ**SUVmax,** Δ**SUVmean, RImax, and RImean) in relation to TRG and (y)pTNM criteria. For these purposes, comparisons of results were performed by Student's* t*-test for unpaired groups. To evaluate the capacity of ^18^F-FDG PET/CT measurements in predicting nCRT response in patients with LRC and to individuate hypothetical cut-off values, ROC curve analysis was performed. A logistic regression model was built to evaluate the predictive capability of the individual ^18^F-FDG PET/CT measurements and their combinations. Statistical evaluation was carried out using SPSS 20.0 for Mac.

## 3. Results

According the Mandard's TRG criterion, the surgical specimen classified 23/56 patients (41.1%) as responders and 33/56 (58.9%) as nonresponders. According to the T parameter of (y)pTNM, the surgical specimen classified 12/56 patients (21.5%) as responders and 44/56 (78.5%) as partial/nonresponders. Tumor characteristics resulting from the histopathologic analysis are reported in Table 2.

### 3.1. Assessment of Response by ^18^F-FDG PET/CT

Sensitivity, specificity, accuracy, PPV, and NPV were 88.6%, 66.7%, 83.92%, 90.7%, and 61.5%, respectively. ^18^F-FDG PET/CT overall parameters are reported in Table 3.

^18^F-FDG PET/CT results regarding TRG showed differences between responders and nonresponders in SUVmax2 (5.22 versus 7.73; *t* = −3.140; *P* = 0.003), SUVmean2 (2.33 versus 3.57; *t* = −3.220; *P* = 0.002), RImax (65.72% versus 52.52%; *t* = 2.278; *P* = 0.027), and RImean (70.18% versus 54.39%; *t* = 2.698; *P* = 0.009) values (Table 4).

^18^F-FDG PET/CT results regarding (y)pTNM showed differences between responders and nonresponders in SUVmax2 (4.17 versus 7.38; *t* = −4.353; *P* = 0), SUVmean2 (1.92 versus 3.38; *t* = −3.976; *P* = 0), RImax (70.32% versus 54.57%; *t* = 2.26; *P* = 0.027), and RImean (73.73% versus 57.37%; *t* = 2.595; *P* = 0.016) values (Table 5).

**Figure 1:**
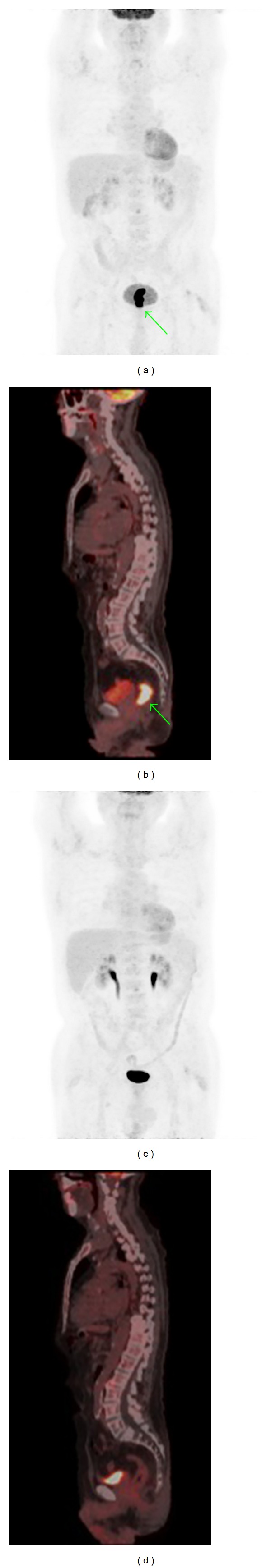
A 77-year-old male with a vegetans eccentric ulcerated lesion, 45 mm in length, localized 3 cm from the anal verge (cT3N0). Baseline ^18^F-FDG PET/CT MIP (a) and sagittal images (b) showed the rectal lesion, with value of 18.9 for SUVmax, 10.4 for SUVmean, and 3.0 SD (green arrows). The post-nCRT ^18^F-FDG PET/CT MIP (c) and sagittal images (d) did not show a pathological uptake of ^18^F-FDG (SUVmax = 2.5, SUVmean = 1.1, and std = 0.3). Histological specimen analysis showed (y)pT0N0 M0, TGR1, and R0 and the patient was classified as a complete responder. In this patient ΔSUVmax, ΔSUVmean, RImax, and RImean results were 16.4, 9.3, 86.77, and 89.42%, respectively.

**Figure 2:**
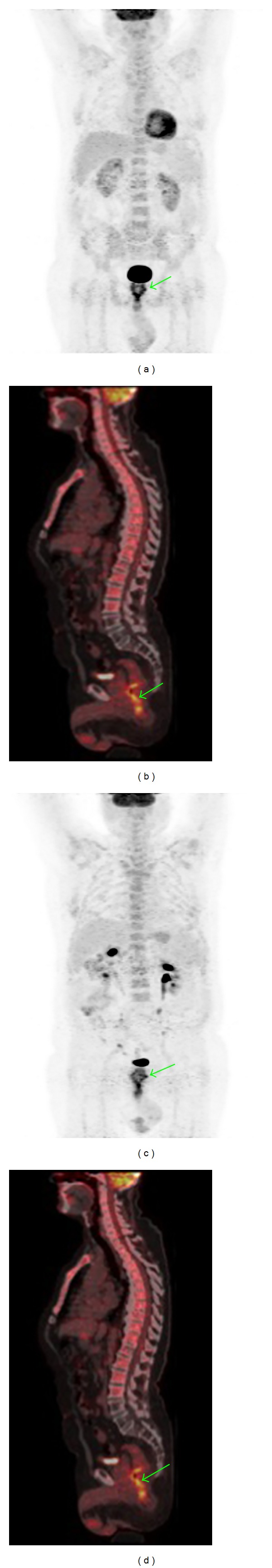
A 76-year-old male with an eccentric lesion, 30 mm in length, localized 2 cm from the anal verge (G3, cT3N0). Baseline ^18^F-FDG PET/CT MIP (a) and sagittal images (b) showed the rectal lesion with a 12.9 value for SUVmax, 5.4 for SUVmean, and 1.5 SD (green arrows). The post-nCRT ^18^F-FDG PET/CT MIP (c) and sagittal images (d) showed, in the same site (green arrows), the persistence of ^18^F-FDG pathological uptake (SUVmax 11.8, SUVmean 5.4, and SD 1.5). Histological specimen analysis showed (y)pT3N0 M0, TGR4, and R0 and the patient was classified as a nonresponder. In this patient ΔSUVmax, ΔSUVmean, RImax, and RImean results were 1.10, 0, 8.5, and 0%, respectively.

Representative images of a responder and a nonresponder patient are shown in Figures 1 and 2, respectively.

### 3.2. ROC Analysis

**Figure 3:**
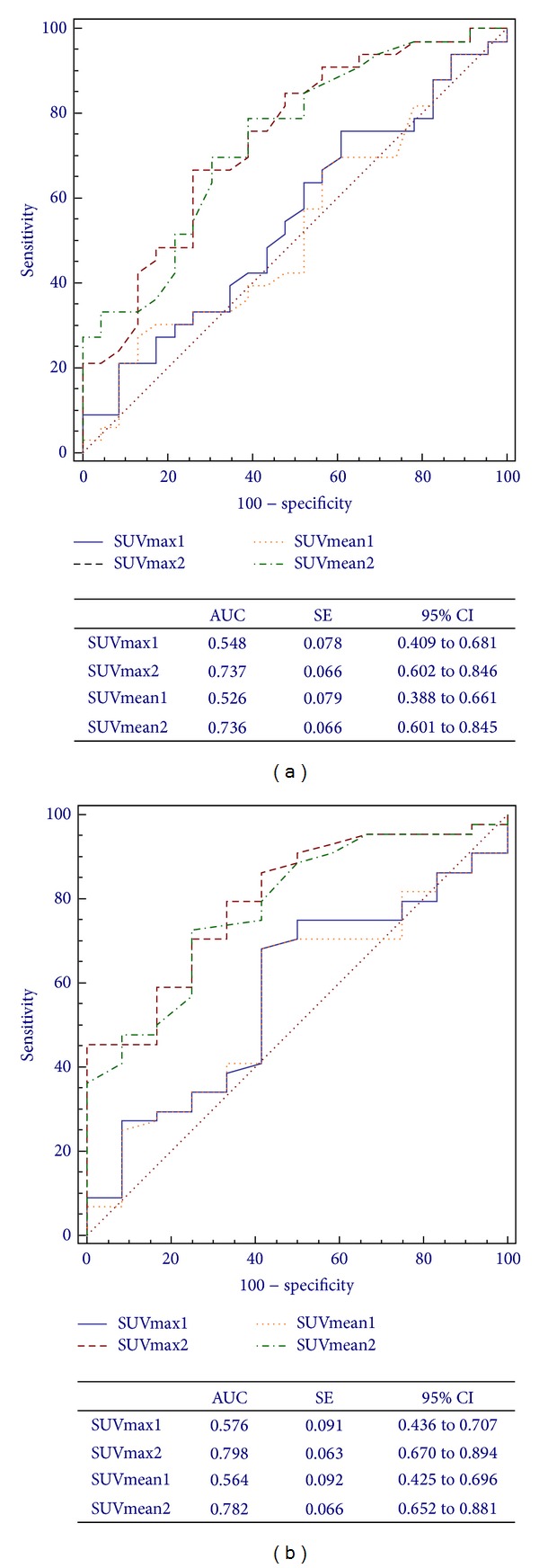
ROC curves for ^18^F-FDG PET/CT assessment of SUVmax1, SUVmean1, SUVmax2, and SUVmean2 in predicting response to CRT based on Mandard's TRG (a) and the (y)pTNM (b) criteria.

Figure 3 shows ROC curve analysis for SUV1 and SUV2 with respect to TRG (Figure 3(a)) and (y)pTNM (Figure 3(b)) response criteria and the corresponding areas under the curves (AUC). SUVmax2 and SUVmean2 showed a better performance in predicting responders with no significant statistical difference between the corresponding SUVmax2 and SUVmean2 AUC (0.737 versus 0.736; *P* = 0.928). The same applies to the (y)pTNM criterion (0.798 versus 0.782; *P* = 0.192).

**Figure 4:**
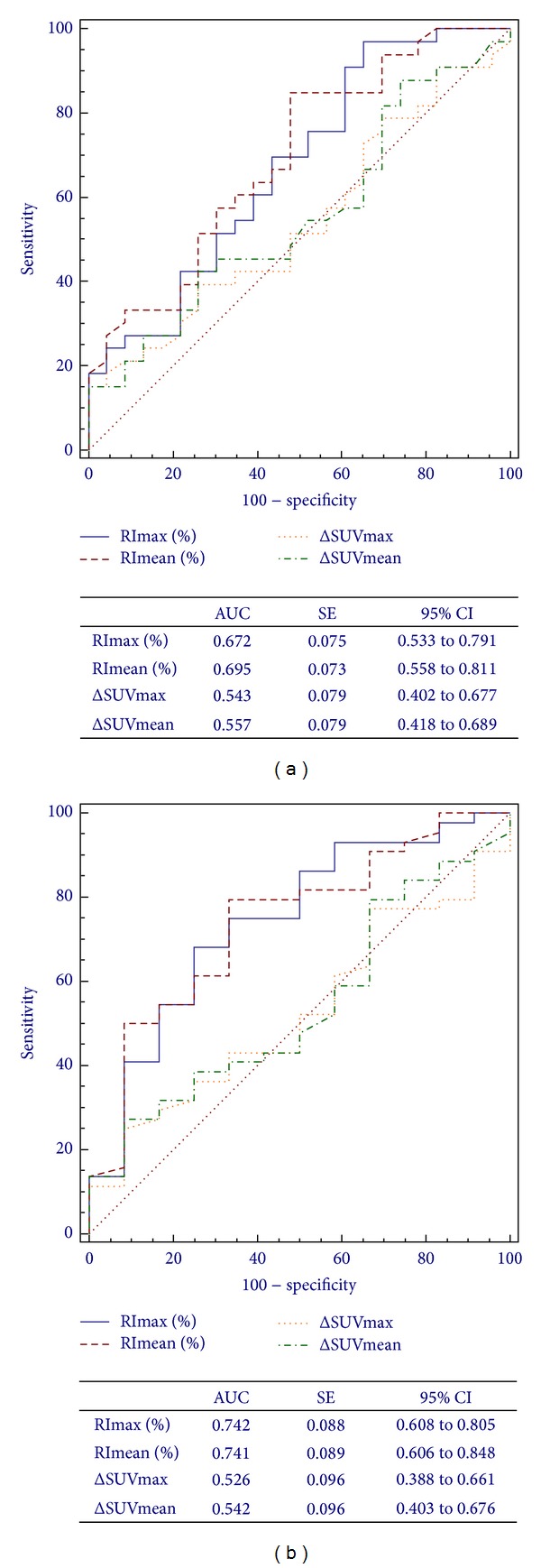
ROC curves for ^18^F-FDG PET/CT assessment of ΔSUVmax, ΔSUVmean, RImax, and RImean in predicting response to CRT based on Mandard's TRG (a) and the (y)pTNM (b) criteria.

Figure 4 shows ROC curve analysis for** Δ**SUV and RI with respect to the TRG and (y)pTNM response criteria. RI values showed a higher AUC than** Δ**SUV, without significant differences between RImax and RImean (0.672 versus 0.695; *P* = 0.292). The same applies to the (y)pTNM criterion (0.742 versus 0.741; *P* = 0.940). In both cases, looking at 95%CI and AUC around 0.5,** Δ**SUV does not appear to be a good predictive tool.

Logistic regression confirmed the predictive role of SUV2; in particular SUVmax2 resulted in the better predictive tool for the (y)pTNM criterion (odds ratio = 1.58) and SUVmean2 for the TRG criterion (odds ratio = 1.87).

Preliminary cut-off values of the most significant parameters (SUV2 and RI), as individuated by ROC curve analysis, are reported in Table 6.

## 4. Discussion

^18^F-FDG PET/CT has a recognized validity for monitoring nCRT effects, but to achieve a correct interpretation of the results appropriate timing is important. Because chemotherapy can produce an inflammatory reaction that lasts for 1 week, while postradiotherapy inflammation may last for 6 months, the choice of interval between the end of treatment and ^18^F-FDG PET/CT is critical. Naturally, the longer the interval, the lesser the probability of obtaining a nonspecific ^18^F-FDG uptake. Nevertheless, waiting for 6 months or more is not clinically justified, especially in patients for whom surgery after nCRT is mandatory [1].

For this reason, in our study all patients underwent ^18^F-FDG PET/CT 5-6 weeks after the end of nCRT and surgery was performed after 8 weeks from the end of the combined treatment, which is not different from the method recommended by the World Health Organization (^18^F-FDG PET/CT scan 7 weeks after nCRT and early surgery 1 week later). In any nCRT for LRC, accurate restaging to assess the success of treatment is critical, as it can guide the optimization of the surgical approach, such as sphincter-saving surgery in deep-seated tumors, less aggressive resection in initially advanced tumors, or the planning of intraoperative radiation therapy depending on tumor response, resulting in an overall enhanced quality of life [3].

Much of the currently reported inaccuracy obtained with purely morphologic modalities has been caused by overstaging because of the inability to distinguish between tumors and radiation-induced inflammation and fibrosis [9]. Numerous previous studies analyzed the role of ^18^F-FDG PET/CT in LRC response to nCRT, but they employed very heterogeneous methods for ^18^F-FDG PET/CT quantification, the evaluation interval, the metabolic response criteria, and the clinical endpoints (histology or survival) [17].

At the current state of the art, Murcia Duréndez et al. achieved better results for ^18^F-FDG PET/CT diagnostic validity than those obtained in previous studies, regardless of whether the authors used visual analysis or a semiquantitative method [8, 18, 19]. The sensitivity and PPV results of our study (88.6% and 90.7%) are as good as those of Murcia, even if the specificity and NPV results were lower (66.7% and 61.5%).

The reported accuracy of ^18^F-FDG PET/CT in determining the responsiveness to nCRT was around 80% in all the studies in the literature, not different from our result (83.9%) [4, 14]. When evaluating the pelvic region (as in patients with LRC), fusion of metabolic and morphological imaging is advantageous to assure a better lesion localization and thus reduce interpretation pitfalls (such as those associated with nonspecific ^18^F-FDG uptake in the bowel lumen, muscles, inflammatory processes, uterine cavity, and brown fat tissue) [20]. However, given the relatively low spatial resolution of PET scanners (about 3–5 mm transaxially at the center of the field of view), ^18^F-FDG PET/CT cannot distinguish major tumor response from complete response [3, 9].

The most important starting point is to perform a baseline ^18^F-FDG PET/CT, before starting therapy. This pretherapy examination must be evaluated both qualitatively and semiquantitatively by comparative SUV [8]. Several PET/CT parameters, including visual-, kinetic-, and SUV-based techniques, have been used as predictors for rectal cancer response to neoadjuvant therapy [9, 10, 21]. The SUVmax is the most commonly studied parameter in the literature for semiquantitative analysis of the glucose metabolism with ^18^F-FDG PET/CT [9, 10]. In quantifications of glucose consumption, the SUVmean has proven to be a stable parameter. For uptake measurements and tracer-kinetic approaches, no difference in accuracy with respect to reproducibility has been reported [21]. It is worth noting that SUVmax measurement is mandatory because its value is the most consistent and less dependent on the ROI size. Nevertheless, because the SUVmax and SUVmean values depend on many other factors (patient weight, interval between FDG administration and image acquisition, and blood glucose level), they must be evaluated carefully to assure a correct interpretation, in particular, when SUVs pre- and posttherapy as well as RI are compared to assess the metabolic response.

A possible explanation for variations in these different parameters for predicting tumor response may be that heterogeneous response criteria have been applied to the previous reports, like the gold standard (complete response, partial response, stable disease, and progressive disease), TRG, and downstaging [12, 22]. Thus, in the present work we evaluated the ^18^F-FDG PET/CT findings with Mandard's TRG criterion of response to neoadjuvant treatment and with the T parameter of (y)pTNM staging.

Grouping TRG1 and TRG2 together as responders is acceptable given the evidence that they have similar prognosis [23]. (y)pT0 corresponds to the absence of neoplastic cells in the surgical specimen, being the unequivocal histopathology parameter for the complete nCRT response, even if it does not describe the modifications after nCRT.

A decade ago, the European Organization for Research and Treatment of Cancer (EORTC) proposed ^18^F-FDG PET/CT criteria for assessing response to treatment according to Mandard's TRG. This proposal has not yet been universally accepted, and 2 main problems remain: (1) to define the timing between the end of therapy and ^18^F-FDG PET/CT and (2) to define the cut-off above which a patient may be considered a responder [1]. In a study of 44 patients, Capirci et al. identified a 66.2% RImax value as the best cut-off value for defining response to therapy and for discriminating responders from nonresponders (according to Mandard's TRG criteria), with 81.2% sensitivity and 79.2% specificity [3]. Subsequently, in a cohort of 81 patients Capirci et al. found similar results [10].

In our study the SUVmax2 cut-off for TRG result was >6.5, with a sensitivity and specificity of 66.7% and 73.9%, respectively, while the RImax cut-off result was ≤78.3% with 93.9% sensitivity and 34.8% specificity. It must be emphasized that the cut-off values identified by all study groups, including ours, are strictly dependent on the patient population analyzed. For this reason, results are quite different in the different studies and cut-off values have to be considered only as a guide and need further validation.

Several studies report a relation of RI with tumor response evaluated by (y)pTNM and tumor regression and response classifications such as responders and nonresponders [12]. Shanmugan et al. reported a complete response rate of 26%, with 58% sensitivity and 78% specificity, using a post-nCRT SUV <4 as the cut-off threshold for predicting (y)pTNM [22]. Kim et al. retrospectively studied 151 patients, analyzing SUVmax results in (y)pTNM responders and nonresponders. In their results SUVmax2 result was 3.03 in responders and 4.49 in nonresponders (*P* < 0.001), while RImax result was 68.16% and 61.35% in responders and nonresponders, respectively. They also indicated a SUVmax2 cut-off >3.55 with 73.7% sensitivity and 63.6% specificity [12].

In our study with a SUVmax2 cut-off >4.3 for predicting (y)TNM, sensitivity and specificity results were 79.5% and 66.7%, respectively. These cut-off values have to be considered with the same caution as those postulated for the TRG response criterion, because they are strictly dependent on our study population and need further validation. However, investigations that adopted the same (y)pTNM criterion reported similar results to those of the present study, namely, that SUV2 is a representative marker of response prediction in rectal cancer patients, for cut-off values ranging from 3.35 to 4.00 [21, 22].

Gadaleta et al. reported no significant difference between the initial SUVmax of responders or nonresponders (*P* = 0.420) with a mean value of the SUVmax reduction of 15.0 ± 7.3%. ROC analysis was performed in order to determine a cut-off value for the SUVmax reduction to discriminate responders from nonresponders (AUC = 0.700; *P* = 0.107). Using a threshold of 36%, ^18^F-FDG PET/CT showed a sensitivity of 100%, a specificity of 60%, PPV of 77%, and NPV of 100% (*P* = 0.002) [13].

Our data suggest that values of SUVmax2, SUVmean2, RImax, and RImean could adequately predict nCRT response by TRG and (y)pTNM criterion. We found SUV2 and RI to be the best predictors for both TRG and (y)pTNM analysis. We did not find any statistical difference between max and mean values, so max values could be easier for physicians to measure.

The use of RImax seems to have the same predictive role of RImean for both TRG and (y)pTNM comparison, so this suggests that SUVmean parameters do not seem to better reflect the nature of the entire tumor mass (viable cells mixed with fibrosis or necrosis) as compared with SUVmax parameters.

Standard criteria for the use of ^18^F-FDG PET/CT in assessing response to nCRT need to be further elucidated. Dual time ^18^F-FDG PET/CT emerges as a valuable tool for the assessment of therapeutic success and to determine whether the response to nCRT in patients with LRC can justify a change in the surgical approach.

We conclude that ^18^F-FDG PET/CT is a reliable technique for evaluating the response to neoadjuvant therapy in LRC. The combination of visual and semiquantitative analysis of the PET/CT data is mandatory even if cut-off values discriminating responders from nonresponders need to be further validated. Therefore, ^18^F-FDG PET/CT should be included in protocols for nCRT response evaluation, even if studies employing identical response criteria and large sample sizes are required to define the most significant parameters for predicting tumor pathologic changes.

## Figures and Tables

**Table 1:** Tumors characteristics at the initial staging.

Mean lesion length	47.2 mm (range 20–100 mm)	
Mean distance between lesion lower edge and anal verge	3.70 cm (range 1–8 cm)	

	*n* (56)	(%)

Characteristics of lesions		
Vegetans	43	76.8
Infiltrans	13	23.2
Eccentric	42	75
Concentric	14	25
Ulcerated	19	33.9
Not ulcerated	37	66.1
Histotypes		
Adenocarcinoma	53	94.6
Mucinous adenocarcinoma	3	5.4
Grading		
G1	6	10.7
G2	26	46.4
G3	14	25
GX	10	17.9
cTNM staging		
II	31	55.4
III	25	44.6
cT		
T2	14	25
T3	42	75
cN		
N0	36	62.3
N1	20	35.7

**Table 2:** Tumors histopathologic characteristics (TNM).

	*n* (56)	(%)
T		
T0	12	21.4
T1	6	10.7
T2	15	26.8
T3	23	41.1
N		
N0	38	67.9
N1	5	8.9
N2	6	10.7
NX	7	12.5
TRG		
I	15	26.8
II	8	14.3
III	19	33.9
IV	13	23.2
V	1	1.8
R (residual cr after resection)		
0	51	91
1	3	5.4
2	2	3.6

**Table 3:** Overall ^18^F-FDG PET/CT parameters.

	Min.	Max.	Mean	SD
SUVmax1	3.8	44.3	18.10	8.86
SUVmean1	1.9	20.3	9.20	4.27
SUVmax2	1.5	12.3	6.70	3.16
SUVmean2	0.6	6.3	3.06	1.53
ΔSUVmax	0.3	37.5	11.40	8.01
ΔSUVmean	0.0	17.40	6.09	4.01
RImax (%)	3.3	88.34	57.95	22.12
RImean (%)	0.0	89.51	60.87	24.24

**Table 4:** ^
18^F-FDG PET/CT results regarding TRG.

	TRG responders	TRG nonresponders	*t*	*P*
	23/56 patients (41.1%)	33/56 patients (58.9%)		
SUVmax1	17.00 (7.91)	18.86 (9.51)	−0.769	0.445
SUVmean1	8.83 (4.09)	9.46 (4.44)	−0.538	0.593
SUVmax2	5.22 (2.84)	7.73 (3.00)	−3.140	0.003
SUVmean2	2.33 (1.29)	3.57 (1.50)	−3.220	0.002
ΔSUVmax	11.78 (7.28)	11.13 (8.58)	0.296	0.768
ΔSUVmean	6.50 (3.76)	5.80 (4.21)	0.633	0.529
RImax	65.72% (18.23)	52.52% (23.21)	2.278	0.027
RImean	70.18% (17.35)	54.39% (26.41)	2.698	0.009

Mean values and standard deviations are reported.

**Table 5:** ^
18^F-FDG PET/CT results regarding (y)pTNM.

	T complete responders	T partial/nonresponders	*t*	*P*
	12/56 (21.5%)	44/56 (78.5%)		
SUVmax1	15.82 (7.11)	18.72 (9.25)	−1.003	0.320
SUVmean1	8.30 (3.60)	9.45 (4.44)	−0.825	0.413
SUVmax2	4.17 (1.98)	7.38 (3.09)	−4.353	0
SUVmean2	1.92 (0.98)	3.38 (1.52)	−3.976	0
ΔSUVmax	11.65 (6.72)	11.33 (8.39)	0.121	0.904
ΔSUVmean	6.37 (3.50)	6.01 (4.17)	0.274	0.785
RImax	70.32 (17.84)	54.57 (22.14)	2.26	0.027
RImean	73.73 (17.60)	57.37 (24.78)	2.595	0.016

Mean values and standard deviations are reported.

**Table 6:** ^
18^F-FDG PET/CT cut-off values for TRG and (y)pTNM response criteria.

Variables	nCRT response criteria	Cut-off	Sensitivity (%)	Specificity (%)
SUVmax2	TRG	>6.5	66.7	73.9
	(y)pTNM	>4.3	79.5	66.7
SUVmean2	TRG	>2.0	78.8	60.9
	(y)pTNM	>2.0	72.7	75
RImax	TRG	≤78.3	93.9	34.8
	(y)pTNM	≤65.1	68.2	75
RImean	TRG	≤74	84.9	52.2
	(y)pTNM	≤74	79.5	66.7

Cut-off values individuated by ROC curve analysis.
